# Linking White-Matter Development to Clinical Variation in Autism: Longitudinal Normative Modelling of FA in the EU-AIMS LEAP Cohort

**DOI:** 10.1101/2025.11.25.690402

**Published:** 2025-11-26

**Authors:** Ramona Cirstian, Natalie J. Forde, Barbora Rehák Bučková, Flavio Dell’Acqua, Tony Charman, Eva Loth, Beth Oakley, Laura Colomar Mollà, Isabel Yorke, Richard Stones, Tobias Banaschewski, Declan G. M. Murphy, Jan K. Buitelaar, EU-AIMS LEAP Group, Christian F. Beckmann, Andre F. Marquand

## Abstract

**Background:**

Autism is characterised by marked neurobiological and clinical heterogeneity, which often limits the sensitivity of traditional group comparisons. Longitudinal normative modelling offers a way to capture heterogeneity and to describe an individual’s brain–behaviour relationships beyond diagnostic labels.

**Methods:**

We analysed diffusion MRI scans from 3 waves of the EU-AIMS Longitudinal European Autism Project (LEAP). Using normative models of white matter fractional anisotropy (FA) we computed individual deviations relative to age typical norms. Our sample included 544 different individuals across all waves (Wave 1: 344 [autistic 189]; Wave 2: 297 [autistic 162]; Wave 3: 226 [autistic 145]), aged 7–37 years, with mean intervals of ∼1.5 years (W1–W2) and ∼6 years (W1–W3). We first tested for group level differences between autism and no-autism and then examined the associations with sensory, adaptive, cognitive, and behavioural measures across development within the autism group.

**Results:**

Although mean FA differences between autistic and non-autistic participants were not significant, correlations between individual differences in FA and behaviour across repeated measurements yielded moderate but reproducible results. The cingulum bundle, uncinate fasciculus, callosal fibres (genu and splenium), and the superior longitudinal fasciculus were among the most frequently identified pathways associated with clinical traits. For these circuits, FA values were higher in individuals with better adaptive functioning (VABS) and lower in those reporting sensory processing (SSP) difficulties. Findings were mixed for associations between FA and symptom severity (ADOS). The largest cross-sectional signal appeared at Wave 2, and although some of the same circuitry re-appeared over about 7.5 years from Wave 1 to Wave 3, effect sizes remained modest and variable, consistent with heterogeneity and power differences across waves, but still pointing to a stable white matter tract pattern.

**Conclusions:**

Autism’s white-matter organisation reflects a dimensional and individually patterned architecture rather than a categorical distinction. Stable, person-specific deviations from shared neurodevelopmental pathways are linked to behavioural variation among autistic individuals. Longitudinal normative modelling therefore provides a practical approach to understand and quantify this heterogeneity.

### Authors

Ramona Cirstian*[1,2], Natalie J. Forde[1,2], Barbora Rehák Bučková[1,2], Flavio Dell'Acqua[3], Tony Charman[3], Eva Loth[3], Beth Oakley[3], Laura Colomar Mollà[3], Isabel Yorke[3], Richard Stones[3], Tobias Banaschewski[4], Declan G. M. Murphy[3], Jan K. Buitelaar[2,5], EU-AIMS LEAP Group, Christian F. Beckmann[1,2,6], Andre F. Marquand[1,2,3]

### Affiliations

1. Donders Centre for Cognitive Neuroimaging, Donders Institute for Brain, Cognition and Behaviour, Radboud University, Nijmegen, the Netherlands
2. Department of Medical Neuroscience, Radboud University Medical Centre, Nijmegen, the Netherlands
3. Institute of Psychiatry, Psychology and Neuroscience, King's College London, UK
4. Department of Child and Adolescent Psychiatry, Central Institute of Mental Health, Medical Faculty Mannheim, Heidelberg University, Mannheim, Germany
5. Karakter Child and Adolescent Psychiatry University Centre, Nijmegen, the Netherlands
6. Wellcome Centre for Integrative Neuroimaging - Oxford Centre for Functional Magnetic Resonance Imaging of the Brain (FMRIB), University of Oxford, UK

**EU-AIMS LEAP Group**
**Acquisition team members:**
*KCL LEAP 3/epilepsy* – Zuzana Suchomelova, Mee Rim Oh, Chirag Mehra, Mei Lin Law, Sanjana Gandhi, Laura Bravo Balsa, Maria Dauvermann, Isabel Yorke, Beth Oakley; *Cambridge* – Rosemary J. Holt, Edward Bullock, Yumnah Kahn, Esme Hayes, Leona Strauss, Noel Lam; *Nijmegen* – Anouk Dykstra, Kim Lamers, Marije Mars, Lucas Geelen, Lotte Beckers, Anna Praat, Feline van Aagten, Sjors Reith, Viola Hollestein, Sanne Kluin, Natalie Forde, Jill Naaijen; *Mannheim* – Anna Kaiser, Sarah Baumeister, Pascal-M. Aggensteiner.
**LEAP consortium members**
Jumana Ahmad, Sara Ambrosino, Bonnie Auyeung, Tobias Banaschewski, Simon Baron-Cohen, Sarah Baumeister, Christian F. Beckmann, Sven Bölte, Thomas Bourgeron, Carsten Bours, Michael Brammer, Daniel Brandeis, Claudia Brogna, Yvette de Bruijn, Jan K. Buitelaar, Bhismadev Chakrabarti, Tony Charman, Ineke Cornelissen, Daisy Crawley, Flavio Dell'Acqua, Guillaume Dumas, Sarah Durston, Christine Ecker, Jessica Faulkner, Vincent Frouin, Pilar Garcés, David Goyard, Lindsay Ham, Hannah Hayward, Joerg Hipp, Rosemary J. Holt, Mark H. Johnson, Emily J. H. Jones, Prantik Kundu, Meng-Chuan Lai, Xavier Liogier D'ardhuy, Michael V. Lombardo, Eva Loth, David J. Lythgoe, René Mandl, Andre Marquand, Luke Mason, Maarten Mennes, Andreas Meyer-Lindenberg, Carolin Moessnang, Nico Mueller, Declan G. M. Murphy, Bethany Oakley, Laurence O'Dwyer, Marianne Oldehinkel, Bob Oranje, Gahan Pandina, Antonio M. Persico, Jack Price, Annika Rausch, Barbara Ruggeri, Amber N. V. Ruigrok, Jessica Sabet, Roberto Sacco, Antonia San Jóse Cáceres, Emily Simonoff, Will Spooren, Julian Tillmann, Roberto Toro, Heike Tost, Jack Waldman, Steve C. R. Williams, Caroline Wooldridge, Iva Ilioska, Ting Mei, Marcel P. Zwiers.

## Abstract

**Background:** Autism is characterised by marked neurobiological and clinical heterogeneity, which often limits the sensitivity of traditional group comparisons. Longitudinal normative modelling offers a way to capture heterogeneity and to describe an individual's brain–behaviour relationships beyond diagnostic labels.

**Methods:** We analysed diffusion MRI scans from 3 waves of the EU-AIMS Longitudinal European Autism Project (LEAP). Using normative models of white matter fractional anisotropy (FA) we computed individual deviations relative to age typical norms. Our sample included 544 different individuals across all waves (Wave 1: 344 [autistic 189]; Wave 2: 297 [autistic 162]; Wave 3: 226 [autistic 145]), aged 7–37 years, with mean intervals of ~1.5 years (W1–W2) and ~6 years (W1–W3). We first tested for group level differences between autism and no-autism and then examined the associations with sensory, adaptive, cognitive, and behavioural measures across development within the autism group.

**Results:** Although mean FA differences between autistic and non-autistic participants were not significant, correlations between individual differences in FA and behaviour across repeated measurements yielded moderate but reproducible results. The cingulum bundle, uncinate fasciculus, callosal fibres (genu and splenium), and the superior longitudinal fasciculus were among the most frequently identified pathways associated with clinical traits. For these circuits, FA values were higher in individuals with better adaptive functioning (VABS) and lower in those reporting sensory processing (SSP) difficulties. Findings were mixed for associations between FA and symptom severity (ADOS). The largest cross-sectional signal appeared at Wave 2, and although some of the same circuitry re-appeared over about 7.5 years from Wave 1 to Wave 3, effect sizes remained modest and variable, consistent with heterogeneity and power differences across waves, but still pointing to a stable white matter tract pattern.

**Conclusions:** Autism's white-matter organisation reflects a dimensional and individually patterned architecture rather than a categorical distinction. Stable, person-specific deviations from shared neurodevelopmental pathways are linked to behavioural variation among autistic individuals. Longitudinal normative modelling therefore provides a practical approach to understand and quantify this heterogeneity.

## 1. Introduction

Autism represents a broad and complex pattern of neurodevelopmental variation affecting communication, behaviour and cognition. First described by Kanner in 1943 (1) as what he termed a “disturbance in human connectedness”, its interpretation evolved through theories characterizing autism from a spectrum of social and communicative differences (2), distinct cognitive processing styles (3) and difficulties in understanding others emotions (4). These perspectives led to the current DSM-5 (5) definition, which recognises autism as as representing a spectrum of diverse social and behavioral characteristics. It is increasingly evident from studies in both neuroimaging and genetics that behavioral definitions only partially describe autism with considerable variation present in biological mechanisms. This suggests that autism reflects a combination of interacting mechanisms rather than a single defining feature (6).

The study of white matter has helped illustrate how abnormal brain connections may be related to various behavioural and cognitive symptoms exhibited in individuals with Autism. Diffusion MRI is a non-invasive technique used to characterise white matter fibres by measuring the movement of water molecules within tissue (7,8). In this context, diffusion tensor imaging (DTI) quantitatively assesses the directional movement of water, specifically fractional anisotropy (FA), which serves as a measure of microstructural organisation (9,10). In early research on Autism, significant FA reductions were reported throughout association fibres connecting anterior and posterior cortical areas (11–13), suggesting atypical connectivity patterns in autism. Later studies broadly supported these findings while also emphasising variability in methodology and development (14), while meta- analysis also demonstrated significant and consistent alterations in language-related fibre tracts (15). Therefore, diffusion imaging of brain white matter is a valuable tool for studying the neural variability contributing to the wide range of behaviours associated with autism.

Autism, although generally considered to be relatively stable across the lifespan, is also grounded in a dynamic developmental course. Autistic characteristics, adaptive functioning, and mental health can change from childhood through adulthood (16,17), with long-term studies reporting variability in diagnostic stability and functional outcomes (18,19). Recognising the need to understand how autism unfolds across the lifespan and how these relate to underlying brain systems, longitudinal paradigms have become central to autism research. Large-scale projects such as the Longitudinal European Autism Project (LEAP) combine prospectively harmonised clinical, cognitive, and neuroimaging data acquisition protocols across childhood, adolescence and adulthood (20,21). Recent multimodal work in LEAP has shown that individual differences in structural and functional brain organisation are linked to dimensional variation in autistic traits (22). Longitudinal studies using structural MRI reveal atypical cortical thinning and non-linear volumetric trajectories (23–25) in autistic participants relative to non-autistic controls, while functional imaging has shown altered maturation of key networks involved in social and cognitive function (26–28). In contrast, diffusion imaging longitudinal studies remain scarce, with only a few studies linking changes in white-matter to differences in core autism-related features (29,30). These findings point to a critical gap in understanding white matter development in autism and its link to clinical features.

Capturing the diversity of developmental trajectories in autism remains a challenge for neuroimaging research. Normative modelling offers a framework for addressing this challenge by quantifying individual brain measure deviations from typical developmental patterns (31,32). Recent methodological advances have made normative modelling a robust tool for studying individual differences in large, heterogeneous datasets. By taking into account confounding factors such as age, sex, and site variation, these models estimate typical brain-behaviour relationships across multi-site neuroimaging data (33–36). Normative models have been used to characterise the diversity of brain structure in autism and other psychiatric and neurodegenerative conditions (37–40). Extending these advances, our previous work: (i) introduced diffusion-based normative models of white-matter FA (41) and (ii) provided a normative modelling framework suited to longitudinal data (42) , providing the foundation for the current study of individual white-matter variation and its relation to clinical features in autism.

The goal of the present study is to characterise individual differences in white-matter development in autism using longitudinal diffusion MRI data from LEAP. Specifically, we aim to (i) quantify individual deviations in white-matter microstructure using diffusion-based normative models of FA within the LEAP cohort; (ii) examine if these deviations differ between the autism and no-autism groups or relate to clinical features within the autism group; and to (iii) assess longitudinal changes in white-matter across LEAP's multiple timepoints, evaluating whether these changes in white matter relate to changes in clinical measures over time. Together, these aims provide a longitudinal framework for linking white-matter organisation with clinical heterogeneity in autism.

Based on the considerable heterogeneity of autism, together with the variability of results from previous diffusion studies, we expected modest, spatially variable associations between white-matter organisation and clinical features, with few robust group-level effects We hypothesised that, at the individual level, deviation scores would be more sensitive than traditional group comparisons and better able to capture individual-specific changes over time. If large effects were present, we anticipated that these would be concentrated in long association and commissural tracts previously implicated in autism (e.g. superior longitudinal fasciculus, corpus callosum) (11,43). Overall, we expected that the main differences would occur at the level of developmental trajectories rather than static, cross-sectional differences.

## 2. Methods

### 2.1. Participants and Study Design

The data were obtained from the EU-AIMS Longitudinal European Autism Project (LEAP), a multicentre programme designed for identifying biological markers of autism across development (20,21). Data were collected at five European centres: the Institute of Psychiatry, Psychology and Neuroscience, King's College London (UK); Radboud University Nijmegen Medical Centre (the Netherlands); the Autism Research Centre, University of Cambridge (UK); University Medical Centre Utrecht (the Netherlands); and the Central Institute of Mental Health, Medical Faculty Mannheim, University of Heidelberg (Germany). Participants with an autism diagnosis and non-autistic controls were recruited using harmonised age- and cognition-based assessments and evaluation protocols to ensure comparability across sites.

For the present analyses, only participants with diffusion MRI data of sufficient quality were included, yielding 544 unique individuals (344 at Wave 1, 297 at Wave 2, and 226 at Wave 3). The mean intervals between waves were approximately 1.5 years (Wave 1–2) and 6 years (Wave 1–3). All participants (or their guardians) provided written informed consent in accordance with local ethics. More details on participation and drop-out across sites and waves are presented in Supplementary Figure 1.A summary of the demographic information is presented in Figure 1, showing age and sex distributions by group per wave.

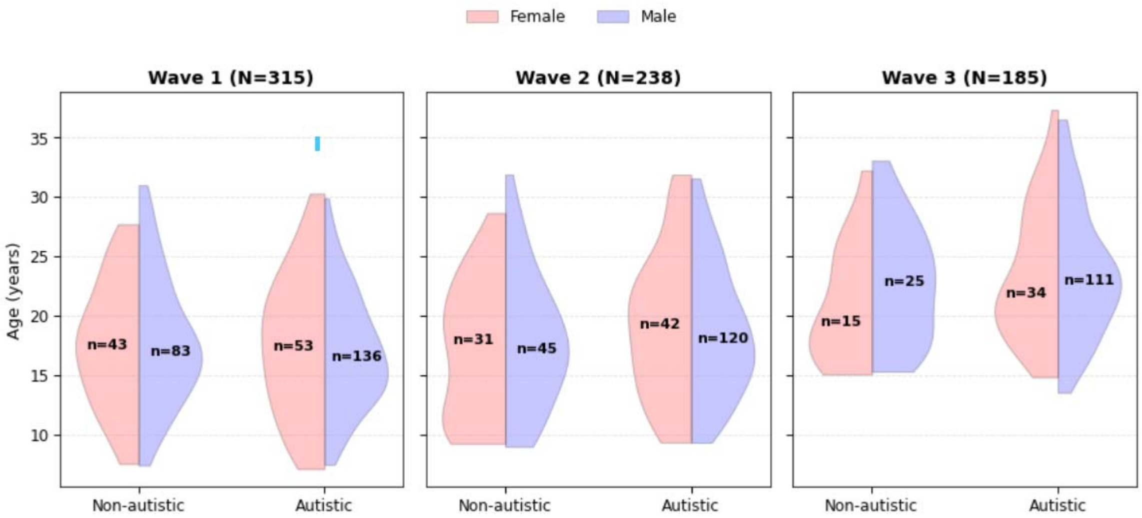

**Figure 1.** Age and sex distributions by diagnostic group. Violin plots show the distribution of participant ages across the three study waves (Wave 1-3) for autism and no-autism groups. Each violin is split by sex (blue = male, red = female).

### 2.2. Diffusion MRI

Single-shell diffusion-weighted data (b = 0/1500 s/mm², 60 directions, 2 mm isotropic voxels) were acquired using echo-planar imaging (EPI) sequences. Preprocessing included noise reduction, motion and eddy-current correction, and susceptibility distortion correction using FSL's eddy (44). Diffusion tensors were fitted with DTIfit to generate FA maps, which were processed using Tract-Based Spatial Statistics (TBSS) (45). Each participant's FA map was projected onto the mean FA skeleton. White matter tracts were segmented using the Johns Hopkins University (JHU) white matter atlas (46), yielding 48 tracts for which mean FA

values were extracted. Full acquisition and preprocessing details are available in Supplementary text 1: Diffusion MRI.

### 2.3. Normative Modelling

We used normative modelling to quantify individual deviations in white-matter organisation relative to population expectations. For each of the 48 tracts in the JHU atlas, pre-trained warped Bayesian Linear Regression models (33) were applied to predict expected FA values conditional on age, sex, and site. To model non-linear age effects, this approach involved applying a third-order polynomial B-spline basis expansion over age, with five evenly spaced knots, combined with a SinhArcsinh warping function.

To harmonise model predictions across sites, small wave-specific adaptation sets of control participants were used to recalibrate normative model intercepts and variance estimates, following transfer-learning principles we have reported and evaluated previously (47). For each wave, the site-matched adaptation sets were assembled from typically developing controls (12 subjects per site), wave unique controls were prioritized while controls within multiple waves were only added when the required number (12) was not met; all remaining participants (autistic and non-adaptation controls) comprised the test sets. Longitudinal normative modelling (42) estimated each subject's change in FA between wave pairs.

Additional quality control (QC) of diffusion data was performed using the same normative modelling framework to detect tract-level artefacts and outliers used in our prior work (48). The QC procedure flagged potential outliers, which were visually inspected; one participant was excluded from Wave 1, while all other data were retained. All analyses were implemented in Python using PCNtoolkit (49). Additional methodological details are described in Supplementary text 2: Normative Modelling.

### 2.4. Clinical Data and Imputation

Clinical and behavioural assessments followed the EU-AIMS LEAP protocol (20,21). For this study, we selected measures consistently available across all three waves: These included the Full-Scale Intelligence Quotient II (FSIQ2), a harmonised IQ estimate derived from the Vocabulary and Matrix Reasoning subtests of Wechsler scales (Wechsler Abbreviated Scale of Intelligence–Second Edition, WASI-II (50); the Autism Diagnostic Observation Schedule, Second Edition (ADOS-2) Social Affect calibrated severity score (SA-CSS), the ADOS-2 Restricted and Repetitive Behaviours score (RRB-CSS), and the ADOS-2 Total Calibrated Severity Score (CSS Total (51,52)); the Social Responsiveness Scale (SRS; (53); the Repetitive Behaviours Scale–Revised (RBS-R; (54)); the Short Sensory Profile (SSP, (55,56)); and the Vineland Adaptive Behaviour Scales, Second Edition (VABS-II), including the Adaptive Behaviour Composite (ABC), Communication, Daily Living Skills, and Socialisation domains (57). Together, these measures capture intellectual ability, autistic symptom severity, sensory reactivity, and adaptive functioning across multiple domains.

To handle incomplete data, we developed a hierarchical, multi-step imputation approach that accounted for structured missingness (design-driven absences, e.g. ADOS administered only to autism participants) and random missingness (incidental loss or incomplete assessments) that we have described previously (58). This is based on the Multivariate Imputation by Chained Equations (MICE) (59) software, iteratively modelling each variable as a function of all others. One-hot encoded subject identifiers were included as fixed effects to account for repeated measurements within participants across waves. The pipeline generated ten imputations to capture uncertainty and enable valid pooled inference. For more details about the missingness assessment and imputation procedure see Supplementary text 3: Missingness and Imputation.

### 2.5. Statistical Analyses

Analyses were performed separately for cross-sectional (Z) and pair-wise longitudinal (Z-diff)(42) scores across the three LEAP waves. Group mean differences were tested for using two-tailed t-tests, while the proportion of extreme deviations ($|Z| > 2.6$; $p \approx 0.01$) per group were compared using Mann–Whitney U-tests for the proportion of extreme cases, with Benjamini–Hochberg false discovery rate (FDR) (60)correction across tracts (n=48).

To examine multivariate brain-behaviour associations, we used penalised canonical correlation analysis (CCA) (61) linking clinical measures and FA deviations using a similar protocol to our prior work (41,61). The analysis was conducted on one of the ten imputed datasets, using only the final hierarchical imputation step. This step followed two earlier levels of missingness estimation, with a random imputation selected at each stage to preserve uncertainty in the imputation process. The resulting clinical dataset for this step included only autistic participants. Fixed and equal sparsity parameters were applied to each view of the data, parameterised such that approxiamtely 50% of features would be selected at each iteration, extracting three canonical components per analysis. Model performance was evaluated out-of-sample through ten train–test (70/30) splits, and significance was determined via 1,000 permutations of the clinical data. Feature stability was quantified as the proportion of splits in which each variable showed non-zero weight; features selected in ≥70% of splits were considered stable. We ran 6 CCAs: 1 for each of the 3 cross-sectional waves and 3 more for the pairwise longitudinal analysis. For longitudinal analyses, Δ-clinical (difference between visit pairs) measures were used along with Z-diff scores.

To dig deeper and help with interpretation of our multivariate findings we conducted univariate correlations between (Δ-)clinical and Z(diff) scores. This was done with each of the ten imputed datasets and combined using Rubin's Rule (62), following the implementation described by Marshall et al. (63), which integrates within- and between-imputation variance to produce unbiased pooled estimates. FDR correction was applied across tracts within each clinical measure. Because several clinical measures showed show moderate skew and sometimes bimodality (especially ADOS – see Figure 2 C), we ran a robustness check on Wave 1 using Spearman's rank correlation which is better suited when variables are skewed or contain outliers (64).Comprehensive statistical details are provided in Supplementary text 4: Statistical Analysis.

## 3. Results

### 3.1. Normative modelling

The normative models were successfully adapted to the LEAP data and fitted across all 48 white matter tracts (JHU) (45), modelling FA trajectories across age (Figure 2A - selected centile plots). The model fits were similar across tracts, with mean explained variance of ~0.44–0.50 for Waves 1–2 and 0.12 for Wave 3. The z-score distributions were close to symmetrical (mean skew ≈ 0) but showed moderate positive kurtosis (≈ 0.8–3.8), indicating slightly heavier tails than would be expected under a perfect Gaussian distribution (See Supplementary Figure S1: Normative Modeling Fit Metrics Summary). It is reasonable that we see less explained variance in this analysis and heavier tailed distributions as compared to our other analyses due to the narrower age range, smaller adaptation set (Wave 1: 36 subjects, Wave 2: 60 subjects, Wave 3: 44 subjects), partial site overlap, and the higher variability typical of paediatric diffusion acquisitions relative to our lifespan cohorts.

### 3.2. Missingness and Imputation of Clinical Data

The proportion of missing data varied with each measure and decreased across waves (as participants dropped out), with Wave 1 having the highest missingness rates and Wave 3 the lowest (Figure 2B). The largest gaps were seen for measures such as the FSIQ2, and SSP, while the VABS and ADOS scales were relatively more complete across waves. None of the variables exceeded the 50 % missingness threshold set for inclusion as further detailed in the Supplementary text 3: Missingness and Imputation . Using the hierarchical MICE procedure in a longitudinal data format along with fixed effects for subjects, we imputed the missing values to avoid discarding incomplete assessments while maintaining within-participant dependency across waves. To validate the imputation, we compared the distribution of the estimated data with that of the observed data, confirming a close alignment in shape while maintaining moderate levels of variation (Figure 2C). Therefore, the imputation procedure addressed the random missing data without introducing any apparent bias or distortion into the clinical profile of the cohort.

### 3.3. Group Comparisons

The analysis across the cross-sectional and longitudinal components showed no significant tract-wise effects remaining once corrected for multiple comparisons. In the cross-sectional waves (1 and 3), Welch's t-tests showed nominal group differences in the Cerebral peduncle (left and right) and the external capsule (right) ($p \approx 0.017$–$0.049$). The Mann–Whitney U (MWU) (65) test also showed nominal differences in the genu and splenium of the corpus callosum, as well as in the anterior limb of the internal capsule (left) ($p \approx 0.015$–$0.036$). In the longitudinal analyses, nominal Z-diff effects appeared in the splenium of the corpus callosum (wave 1–3; Welch's t $p = 0.0289$) and in the cerebral peduncle (left and right) and posterior corona radiata (left) (wave 2–3; $p = 0.013$–$0.045$), with MWU nominal differences in the splenium of the corpus callosum, retrolenticular part of the internal capsule (right) (wave 1–3; $p \approx 0.011$–$0.017$), and the cerebral peduncle (left) (wave 2–3; $p = 0.009$). However, none of these findings were significant after correcting for multiple tests. Details are presented in the Supplementary Table 1: Group comparison results.

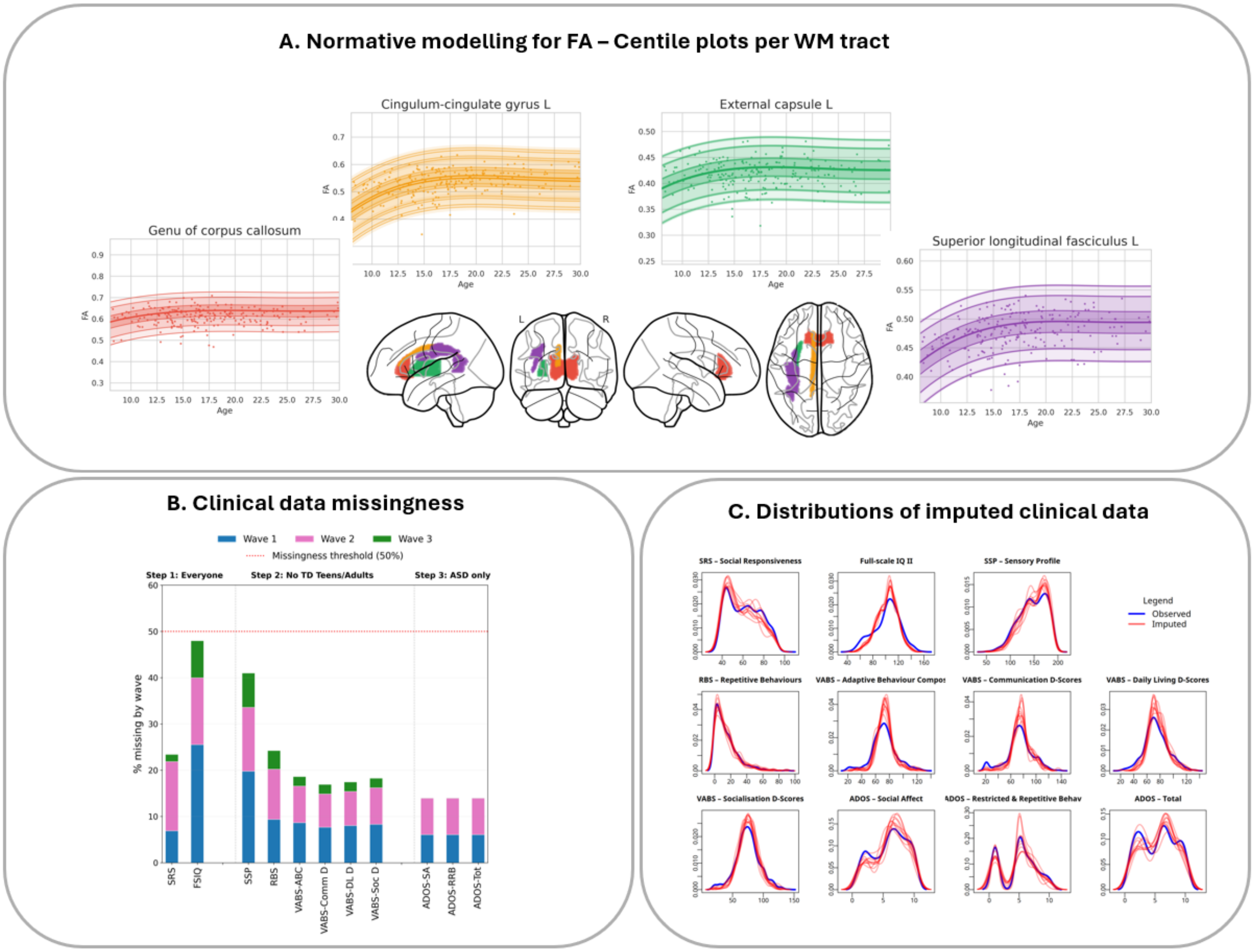

**Figure 2. Normative modelling and clinical data completeness**. (A) *FA normative models.* Centile curves from Wave 1 are shown for white matter tracts which recurred throughout the CCA and correlation analyses, with shaded areas representing the 5th–95th percentile range. Individual data points indicate observed FA values across participants. (B) *Clinical data missingness.* Percentage of missing values per variable and wave, shown for each imputation step. (C) *Imputed clinical data distributions.* Density plots compare observed (blue) and ten imputed (red) values across all clinical measures, showing mostly overlapping distributions and suggesting that the imputed values approximate the observed data well.

### 3.4. Canonical correlation analysis (CCA)

Cross-sectional CCA results are summarized in Figure 3 and reveal significant out-of-sample correlations between white matter FA deviations and the clinical measures for Waves 1 and 2 but not Wave 3 within the autism group. Significance and robustness was established were evaluated using 10 repeated train–test splits and 1,000 permutations of the clinical data. In Wave 1, the first component shows an out-of-sample correlation of $r = 0.26 \pm 0.11$ with $p = 0.001$, consistently selecting FA loadings in the cingulum L, anterior limb of the internal capsule R, external capsule L, and genu of the corpus callosum for the first component. Wave 2 showed a similar pattern (C1: $r = 0.32 \pm 0.10$, $p = 0.001$), consistently selecting the bilateral cingulum, superior longitudinal fasciculus, splenium of the corpus callosum, and cerebral peduncles for the first component. Although no significant components were found in Wave 3 (all $p \geq 0.05$), the stability analysis still highlighted several of the same FA tracts as in the earlier waves, with the superior longitudinal fasciculus, cingulum, and external capsule again showing stable loadings. None of the clinical variables showed consistently selected loadings across splits for any of the cross-sectional CCA analysis.

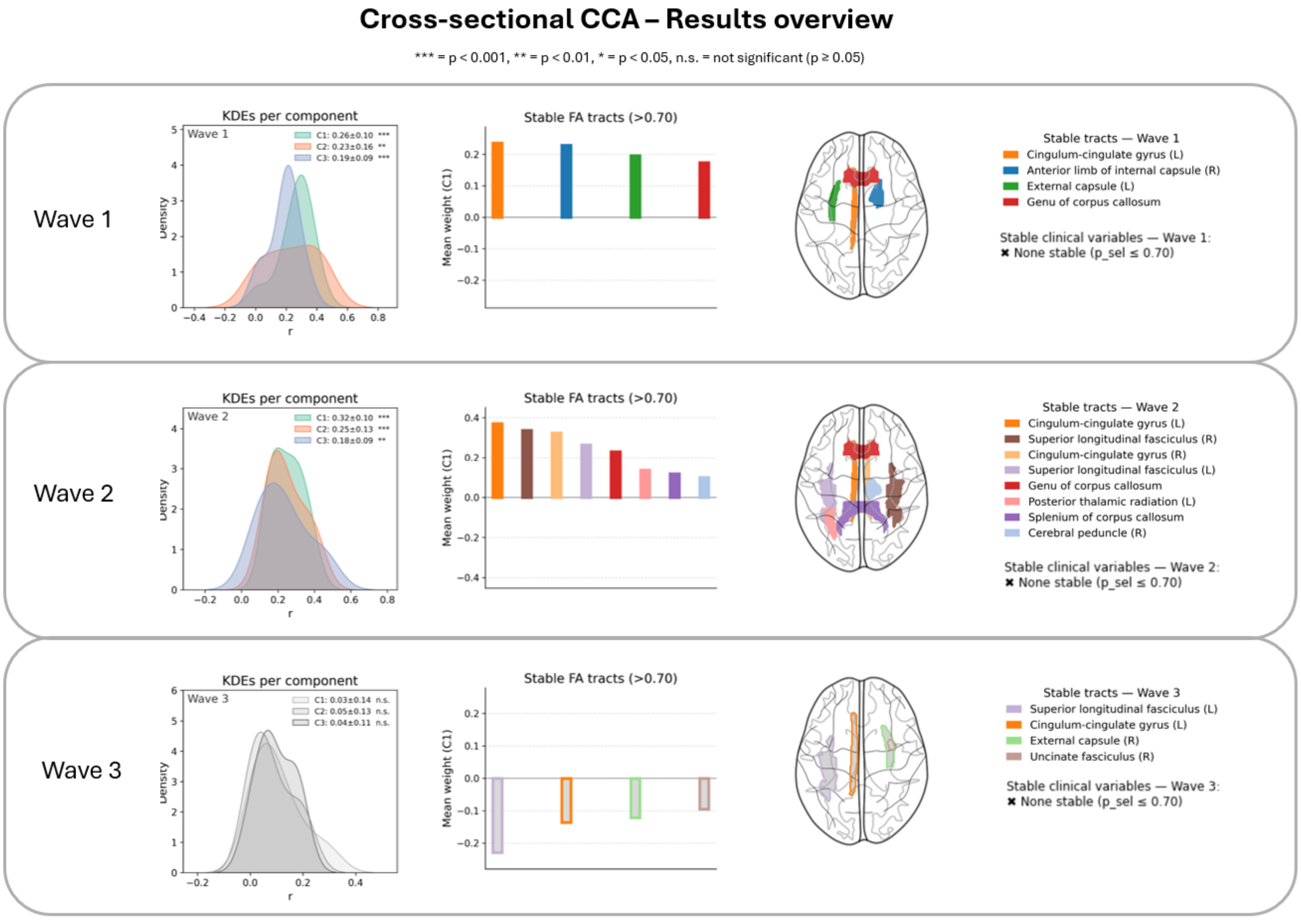

**Figure 3. Cross-sectional CCA - Results overview.** Kernel density plots (left) show the distribution of canonical correlations across 10 out-of-sample data splits, with significance determined by permutation testingBar plots (middle) depict mean component weights for stably selected tracts in the first component, and brain maps (right) illustrate their spatial distribution. Coloured density curves and bars indicate significant components, whereas the grey represent non-significant components shown for comparison. Robust components were identified in Waves 1 and 2, driven by consistent white matter loadings in commisural and long association tracts, whereas no significant or stable modes were detected in Wave 3. No stably selected clinical loadings were identified for any wave.

Across the longitudinal analyses, only the Wave 1-3 comparison had significant canonical correlations. C1 showed a moderate association ($r = 0.36 \pm 0.17$, $p = 0.001$) and included stable FA Z-diff loadings in several projection and association tracts, such as the genu of the corpus callosum, left inferior cerebellar peduncle, bilateral anterior corona radiata, right sagittal stratum, bilateral external capsule, left uncinate fasciculus, and right tapetum. This was also the only analysis in which clinical variables were stably selected, specifically ΔCSS total and ΔSA-CSS. The Wave 1-2 and Wave 2-3 intervals did not show significant results (C1: $r = -0.12 \pm 0.15$, $p = 0.87$; and $r = 0.16 \pm 0.24$, $p = 0.10$ respectively), though each still had a few stable tract loadings. These involved the left cingulum–hippocampal tract and right posterior thalamic radiation for Wave 1-2, and the splenium of the corpus callosum, inferior and cerebral peduncles, anterior corona radiata, cingulum–cingulate gyrus, fornix cres/stria terminalis, and superior fronto-occipital fasciculus for Wave 2-3. No stable clinical variables were identified in either of these pairs.

**Figure 4. Longitudinal CCA – results overview.** Density plots (left) show the distribution of canonical correlations across 10 out-of-sample splits, bar plots (middle) illustrate mean weights for stably selected FA Z-diff tracts from the first component, and brain maps (right) display their spatial distribution. The analysis relates changes in white-matter FA (Z-diffs) to changes in clinical scores (Δ-clinical measures). Coloured density curves and bars indicate significant components, whereas the grey represent non-significant components shown for comparison. For the Wave 1-3 interval, all three canonical components were significant, and the first component showed stable loadings in a range of projection and association tracts together with two stably selected clinical variables (ΔCSS total and ΔSA-CSS). The Wave 1-2 and Wave 2-3 intervals did not show significant correlations, although each included a number of stably selected tracts. No clinical variables were stably selected in these pairs.

### 3.5. Pooled Pearson Correlation Analysis

To identify the univariate associations with symptoms within the autism group. Pearson correlations were computed and pooled across ten imputations using Rubin's rules (62), following the implementation described by Marshall et al. (63),to account for uncertainty, with FDR correction applied within the clinical family.

Because several clinical variables showed non-normal distributions, we also computed Spearman correlations (66) for the Wave 1 data. Spearman identified more FDR-significant associations than Pearson (57 vs 33), but most results overlapped (84.8%) and showed consistent directions. This suggests that the main pattern of findings was stable and not dependent on the choice of correlation method.The cross-sectional analyses showed significant associations between Z-scores and multiple clinical dimensions across waves (Figure 5, left panel). In Wave 1, sensory processing scores (SSP) and restricted behaviours (ADOS-RRB) showed widespread negative associations, while adaptive communication (VABS) exhibited positive correlations, particularly in cerebellar and fronto-limbic tracts. Several of these effects survived FDR correction, including in the uncinate fasciculus, cerebellar peduncles, and cingulum. Wave 2 showed widespread correlations between VABS and many of the same tracts from in Wave 1 but also parts of the internal capsule, thalamic radiation and sagittal stratum.  By Wave 3, associations persisted but were weaker and less robust after correction.

Longitudinally, we observed less pronounced effects, with only a few nominal associations emerging between changes in clinical scores and Z-diff scores (Figure 5, right panel). Wave 2-3 comparisons showed nominal correlations in ADOS scores across cingulate and projection pathways, with one tract (left superior fronto-occipital fasciculus) surviving FDR correction. Moderate ADOS correlation patterns were also seen in Wave 1-3 for fronto-limbic and thalamic pathways. The pontine crossing tract emerged as a key region for longitudinal change.

Overall, these findings suggest that associations between white matter micostructure and clinical features in autism are broadly consistent across timepoints, particularly in fronto-limbic and cerebellar regions, but show reduced sensitivity in longitudinal analyses, possibly due to much smaller sample size.

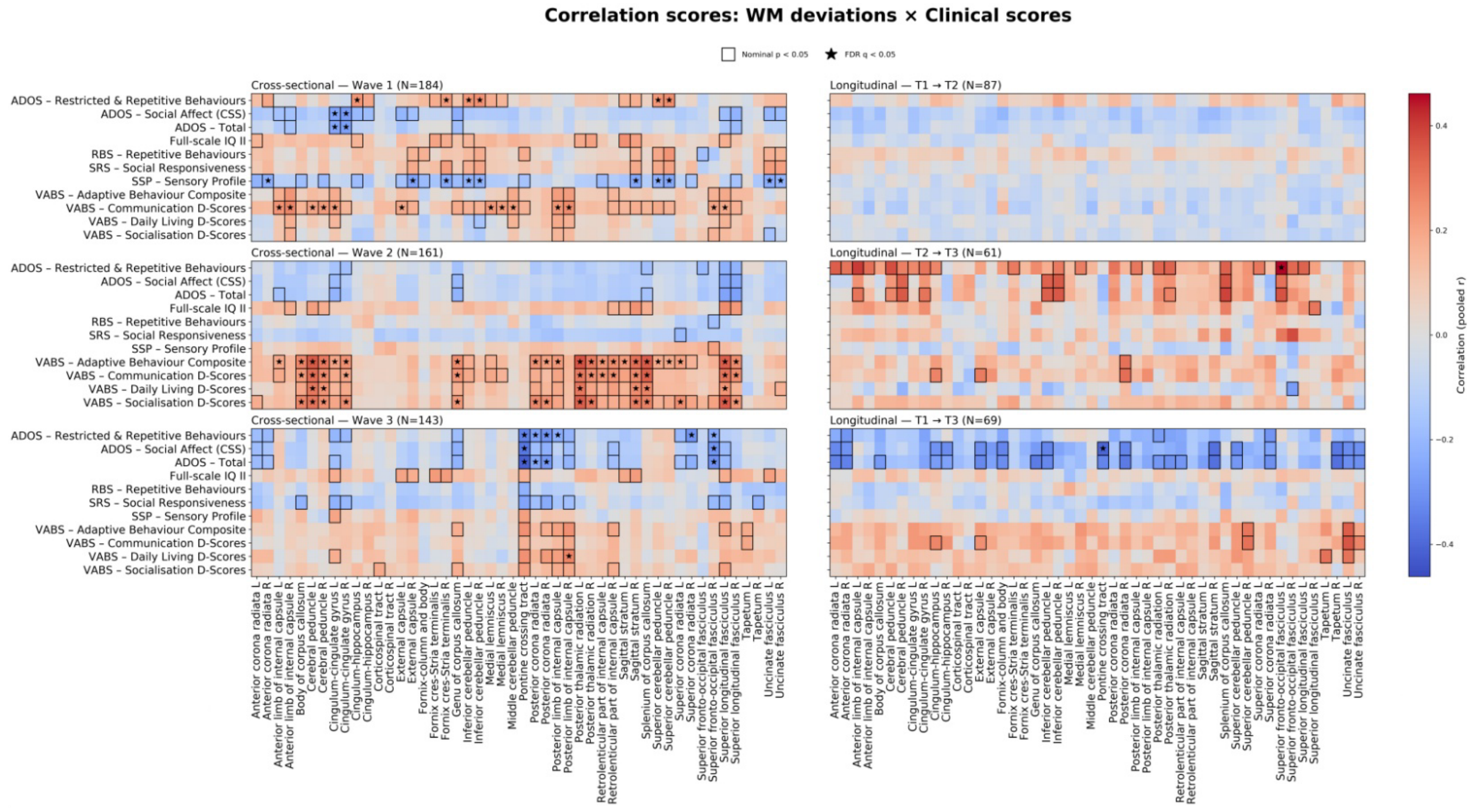

**Figure 5.** Pearson correlations between white matter deviations and clinical scores, pooled across 10 imputations using Rubin's rules. Left: Cross-sectional results for each wave. Right: Longitudinal associations between wave pairs, relating FA Z-diff to Δ-clinical scores.

## 4. Discussion

In this study, we used normative modelling to characterise how white-matter organisation relates to continuous behavioural traits in autism, both cross-sectionally and over time. By modelling individual deviations from lifespan reference patterns rather than relying on group averages, we showed that autism aligns more closely with a dimensional rather than a categorical model of brain organization. We found that meaningful individual deviations, rather than group-mean differences, accounted for variance in clinical traits within fronto-limbic, callosal, and cerebellar–thalamo-cortical circuits. White matter–behaviour relationships were reproducible across waves, with adaptive, sensory, and behavioural measures mapping onto overlapping but directionally distinct pathways. Longitudinal analyses further showed that autism-related circuits are relatively stable, as the same fronto-limbic and cerebellar systems continued to link changes in brain structure with changes in behaviour.

Our first conclusion is that white matter alterations in autism are of a dimensional nature and are not entirely associated with diagnosis. Using normative modelling we were able to reveal significant correlation between individual deviations in white matter integrity and clinical traits in autism. This finding is supported by the CCA results showing stable component loadings in the cingulum, corpus callosum, and cerebellar tracts across Waves 1 and 2, by the pooled correlations revealing direction-specific associations between FA deviations and sensory (SSP), adaptive (VABS), and behavioural (ADOS) traits; and by the absence of significant group-mean FA differences despite clear within-group dimensional effects. Therefore, we demonstrate that autism is characterised by stable yet individually patterned white matter organisation, consistent with the broader view of neurodevelopmental diversity as a continuum rather than a dichotomy.

Recent neuroimaging research has shifted focus towards individualized, dimensional models. Using normative modelling, Zabihi et al. (37,67) demonstrated that autistic brains are characterized by considerable individual deviation in cortical thickness, showing only minimal group-level differences, revealing clinically relevant subtypes characterized by both increases and decreases in cortical thickness. In the same context, Bethlehem et al. (68) found that normative models of cortical thickness accurately predicted behavioural variation in autism, suggesting that categorical group differences may obscure meaningful signal by averaged comparisons. At the white-matter level, Aoki et al. (43) showed that associations between reduced FA were widespread and distributed throughout both callosal and projection tracts, consistent with our own observations that dimensional effects cluster within shared networks rather than discrete diagnostic boundaries. Evidence from Rødgaard et al. (69) further supports this view: as sample size has increased, effect size between autistic/control groups has diminished; indicating that increased diagnostic heterogeneity can disperse categorical contrasts and strengthen the rationale for directly modeling individual variation.

Second, we show that the white matter pathways associated with clinical traits in autism are stable across development. We see consistent patterns resulting from our analyses: Vineland Adaptive Scores (specifically communication and total subscale scores) correlate with tracts such as the cingulum, corpus callosum, internal capsule and cerebellar peduncles; however, ADOS scores display both mixed and recurrent associations, positive for restricted behaviors and negative for social domains, in the fronto-limbic and cerebellar regions. These same patterns are observed at all developmental stages and waves, even though the statistical significance varies due to variations in sample size. This shows that while each person's brain profile is unique, certain neurostructural signatures for sensory versus adaptive functioning do replicate consistently.

Our findings align with a growing body of research showing that white-matter differences in autism relate more to individual traits than to diagnostic categories. The relationship between autism trait severity and changes in limbic and emotion-related tracts reported by Gibbard et al. (70) is in line with our findings in the cingulum and uncinate fasciculus. The correlation between sensory processing and FA in association and limbic tracts described by Pryweller et al. (71) also matches the negative sensory correlations we observed in Wave 1. Finally, Ni et al. (72) showed that self-regulation, rather than diagnosis alone, accounts for variability in white-matter structure within individuals, further supporting a dimensional view of brain–behaviour relationships.

Third, autism-related white matter circuits show longitudinal stability, linking brain and behavioural change across development. Using white matter microstructural differences as markers for clinical trait changes and comparing these across waves, we demonstrated that the same fronto-limbic, callosal and cerebellar-thalamo-cortical systems were also significant longitudinally. By using this difference-vs-differences approach, Wave 1-3 pair shows that tracts including the inferior cerebellar peduncle, genu of the corpus callosum, external capsule and anterior corona radiata remained associated with behaviour change, providing evidence that these circuits do not represent transient findings, but an ongoing relationship between evolving brain structure and autistic traits over time.

At tract level, recent studies show patterns consistent with ours. Tung et al. (73) have shown similar white matter variability within the prefrontal regions of the corpus callosum and the long association fibers, consistent with our finding using normative models. Zhang et al. (30) have demonstrated that lower FA in the superior longitudinal fasciculus is associated with increased communication difficulty and that FA values in the superior thalamic radiation are good predictors of diagnostic outcome. Similarly, our findings show that higher Vineland adaptive scores were linked to stronger FA in association and thalamo-cortical tracts. Andrews et al. (29) also demonstrated that slower FA development in the cingulum, superior longitudinal fasciculus, internal capsule and splenium correlated with changes in ADOS severity scores, supporting the brain-behavior relationships observed in our longitudinal analysis, pointing to stable fronto-limbic and callosal circuits as developmental anchors.

There are several important methodological factors to consider. The multi-view sparse canonical correlation analysis allowed us to establish an association between white matter abnormalities and clinical features using a reliable and interpretable methodology. Given the modest sample size, we chose to fix our sparsity parameters for all analyses to avoid instability associated with nested cross-validation. Additionally, we implemented permutation testing as well as stability selection (74), to provide stable identification of the relevant features.

Sample size and data completeness pose an important limitation. Greater overlap across waves and reduced clinical data missingness would improve longitudinal power and interpretability. Although multiple imputations go some way to addressing this, they do not replace truly observed data. Nevertheless, this approach offers a robust and principled way to estimate missing values while preserving sample size and maintaining statistical power.

In this study we demonstrated extensive associations between individual variation in white-matter organisation and autism clinical features, in the absence of group-level differences between autistic and non-autistic individuals. This suggests that white matter alterations in autism follow a dimensional and developmentally stable pattern. Using normative modelling and multivariate analysis, we found that individual deviations within shared fronto-limbic, callosal, and cerebellar-thalamo-cortical circuits provide the strongest link to adaptive, sensory, and behavioural traits. Rather than reflecting categorical group differences, these findings point to consistent yet individually patterned white-matter architecture across development. While larger and more complete longitudinal datasets will be essential to deepen this understanding, our results offer a clear framework for studying heterogeneity in autism.

## Abbreviations

ABC — Adaptive Behaviour Composite
ADOS — Autism Diagnostic Observation Schedule
ASD — Autism Spectrum Disorder
B-spline — Basis spline
BLR — Bayesian Linear Regression
CCA — Canonical Correlation Analysis
CSS Total —Calibrated Severity Score (ADOS Total)
DSM-5 — Diagnostic and Statistical Manual of Mental Disorders, Fifth Edition
DTI — Diffusion Tensor Imaging
DTIFIT — Diffusion Tensor Imaging Fitting tool (FSL)
DWI — Diffusion-Weighted Imaging
FA — Fractional Anisotropy
FDR — False Discovery Rate
FSIQ2 — Full-Scale Intelligence Quotient, two-subtest form
FSL — FMRIB Software Library
LEAP — Longitudinal European Autism Project
MICE — Multivariate Imputation by Chained Equations
MRI — Magnetic Resonance Imaging
msCCA — Multi-view sparse Canonical Correlation Analysis
QC — Quality Control
RBS — Repetitive Behaviours Scale
RRB-CSS — Restricted and Repetitive Behaviours Calibrated Severity Score
SA-CSS — Social Affect Calibrated Severity Score
SRS — Social Responsiveness Scale
SSP — Short Sensory Profile
TBSS — Tract-Based Spatial Statistics
VABS — Vineland Adaptive Behaviour Scales
Z — Normative deviation score
Z-diff — Normative deviation of longitudinal change

## Acknowledgments

### Funding

This research was supported by grants from the European Research Council (ERC, grant “MENTALPRECISION ”10100118) and NIH grant number 1R01MH130362-01A1.

### Author contributions:

Conceptualisation: RC, NF, AM, CB
Methodology: RC, NF, AM, BRB
Validation: RC, NF, AM,
Software: RC
Formal analysis: RC, NF, AM
Visualisation: RC
Supervision: NF, AM, CB
Writing—original draft: RC
Writing—review & editing: RC, NF, AM, CB
Resources: FD, TC, EL, BO, LCM, IY, RS, TB, DM, JB, EU-AIMS LEAP Group

### Financial Disclosure or Competing interests:

CB is director and shareholder for SBGneuro
None of the other authors report any biomedical financial interest or potential conflicts of interest.

### Data and materials availability:

The data that support the findings of this study are available from EU-AIMS LEAP which were used under licence.
All scripts used for obtaining the results presented in this paper are publicly available at: https://github.com/ramonacirstian/leap_fa_normative_modelling
